# Association of Perceived Neighborhood Health With Hypertension Self-care

**DOI:** 10.1001/jamanetworkopen.2022.55626

**Published:** 2023-02-10

**Authors:** Joseph Lunyera, Clemontina A. Davenport, Patti Ephraim, Dinushika Mohottige, Nrupen A. Bhavsar, Maya N. Clark-Cutaia, Ashley Cabacungan, Nicole DePasquale, Sarah Peskoe, L. Ebony Boulware

**Affiliations:** 1 Division of General Internal Medicine, Department of Medicine, Duke University School of Medicine, Durham, North Carolina; 2 Department of Biostatistics and Bioinformatics, Duke University School of Medicine, Durham, North Carolina; 3 Feinstein Institutes for Medical Research, Northwell Health, Manhasset, New York; 4 Division of Nephrology, Department of Medicine, Duke University School of Medicine, Durham, North Carolina; 5 Rory Meyers College of Nursing, New York University, New York

## Abstract

**Importance:**

Hypertension self-management is recommended for optimal blood pressure (BP) control, but self-identified residential contextual factors that hinder hypertension self-care are understudied.

**Objective:**

To quantify perceived neighborhood health and hypertension self-care and assess interactions with the area deprivation index (ADI) and healthy food availability at home.

**Design, Setting, and Participants:**

A cross-sectional study was conducted in Baltimore, Maryland, including primary care adults enrolled in the Achieving Blood Pressure Control Together trial between September 1, 2013, and June 30, 2014. Participants were Black and had at least 2 BP readings greater than or equal to 140/90 mm Hg in the 6 months before enrollment. Analyses were conducted from August 5, 2021, to January 28, 2022.

**Exposures:**

Participants’ perceived neighborhood health, defined as the mean standardized score across 4 subdomains of aesthetic quality, walkability, safety, and violence, with a higher score signifying better neighborhood health.

**Main Outcomes and Measures:**

Hypertension self-care behavior and self-efficacy. Multivariable generalized linear models were fit regressing each outcome on perceived neighborhood health (higher scores on each domain signify better perceived neighborhood health), adjusted for confounders, and interaction terms between neighborhood health and potential modifiers (ADI [higher percentiles correspond to more deprivation] and healthy food availability [higher scores indicate greater availability]) of the primary association were included.

**Results:**

Among 159 participants (median [IQR] age, 57 [49-64] years; mean [SD] age, 57 (11) years; 117 women [74%]), median (IQR) hypertension self-care behavior was 50 (45-56) and self-efficacy was 64 (57-72). Better perceived neighborhood health was associated with greater hypertension self-care behavior (β, 2.48; 95% CI, 0.63-4.33) and self-efficacy (β, 4.42; 95% CI, 2.25-6.59); these associations persisted for all neighborhood health subdomains except aesthetic quality. There were no statistically significant interactions between perceived neighborhood health or its subdomains with ADI on self-care behavior (*P* = .74 for interaction) or self-efficacy (*P* = .85 for interaction). However, better perceived neighborhood aesthetic quality had associations with greater self-care behavior specifically at higher healthy food availability at home scores: β at −1 SD, −0.29; 95% CI, −2.89 to 2.30 vs β at 1 SD, 2.97; 95% CI, 0.46-5.47; *P* = .09 for interaction). Likewise, associations of perceived worse neighborhood violence with lower self-care behavior were attenuated at higher healthy food availability at home scores (β for −1 SD, 3.69; 95% CI, 1.31-6.08 vs β for 1 SD, 0.01; 95% CI, −2.53 to 2.54; *P* = .04 for interaction).

**Conclusions and Relevance:**

In this cross-sectional study, better perceived neighborhood health was associated with greater hypertension self-care among Black individuals with hypertension, particularly among those with greater in-home food availability. Thus, optimizing hypertension self-management may require multifaceted interventions targeting both the patients’ perceived contextual neighborhood barriers to self-care and availability of healthy food resources in the home.

## Key Points

**Question:**

Is perceived neighborhood health associated with hypertension self-care behavior or self-efficacy?

**Findings:**

In this cross-sectional analysis of data from a randomized clinical trial, better perceived neighborhood health was associated with greater hypertension self-care behavior and self-efficacy. The magnitude of these associations was higher among participants with more healthy food availability at home.

**Meaning:**

The findings of this study suggest that perceived contextual neighborhood features and in-home resources might play a role in hypertension self-care and could be targeted in interventions for optimizing hypertension self-management.

## Teaser

This cross-sectional study examines hypertension self-management in Black individuals in socially disadvantaged settings.

## Introduction

Hypertension self-management is recommended in the most recent clinical practice guidelines for achievement of optimal blood pressure (BP) control.^1^ Self-management of hypertension, or the capacity to engage in behaviors necessary to achieve optimal BP control, entails healthy diet, physical activity, no smoking, taking prescribed BP medications, and self-monitoring of BP^2^ and can be assessed using validated instruments, such as the Hypertension Self-Care Profile.^3^ Prior studies have established the effectiveness of hypertension self-management on improved BP control.^4,5,6,7^ However, contextual factors that hinder hypertension self-management, particularly among individuals with social disadvantages, are not fully understood,^4^ limiting scalability and thus a broad impact of effective, contextually sensitive interventions.

Residential environments, both outside (ie, neighborhood) and within the home (eg, foods resources), could play an important role in patients’ hypertension self-management. The residential environment could promote hypertension self-management by providing a safe space for physical activity and venues for obtaining affordable, healthy food. Prior studies have predominantly used socioeconomic measures, such as the area deprivation index (ADI), to assess the role of the neighborhood built environment on risk and management of hypertension.^8,9,10,11,12^ However, emerging data suggest that neighborhood socioeconomic characteristics might not capture the full range of contextual factors that hinder patients’ hypertension self-management. Patient-described factors might play an additional role, as suggested in a recent study in which the association of neighborhood socioeconomic status with BP control became nonexistent after accounting for patient characteristics.^11^ To our knowledge, studies have not investigated the association between patients’ perceived neighborhood characteristics, such as aesthetic quality, and hypertension self-management.^12,13^ One prior study suggested that individual-level socioeconomic status modifies the association of the neighborhood built environment with health.^14^ However, whether perceived neighborhood health and hypertension self-care differ by neighborhood-level socioeconomic status (eg, as measured by ADI) or the in-home environment, such as availability of foods (eg, fruits and vegetables) and kitchen appliances (eg, oven and refrigerator) that are often integral for food preparation, is unclear. Characterizing these potential synergistic effects could aid efforts to identify contextual roadblocks that might be addressed in future interventions to maximize BP control, particularly among individuals with social disadvantages who may have disproportionately suboptimal BP control compared with others.

We sought to study the association of perceived residential neighborhood environments with hypertension self-management. We hypothesized that better perceived neighborhood health would be associated with greater hypertension self-management. Because neighborhood deprivation is linked with poor BP control,^11^ we hypothesized that the magnitude of this association would be greater among individuals with higher vs lower ADI levels. We also hypothesized that the magnitude of the association of perceived neighborhood health with hypertension self-care would be higher among participants with greater healthy food availability at home. We used data from the Achieving Blood Pressure Control Together (ACT) trial to evaluate these hypotheses.

## Methods

### Study Design and Population

The parent ACT trial was a randomized comparative effectiveness trial (NCT01902719).^15^ Participants in the ACT trial were Black or African American individuals with social disadvantages, English-speaking adults (≥18 years) who received care at an academically affiliated community-based primary care clinic in East Baltimore, Maryland; had 2 blood pressure readings greater than or equal to 140/90 mm Hg in the 6 months prior to enrollment; and planned to maintain a stable residential address for in-person home visits by the study team. Participants were recruited and enrolled between September 1, 2013, and June 30, 2014. The data for the parent ACT trial were collected in person at the study site, through telephone surveys, and via in-person visits at participant homes. The ACT trial, with the present study part of the protocol, received approval from the Johns Hopkins University School of Medicine Institutional Review Board, and all participants provided written informed consent prior to trial enrollment; participants did not receive financial compensation. The reporting of this cross-sectional study of the ACT trial follows the Strengthening the Reporting of Observational Studies in Epidemiology (STROBE) reporting guideline for the reporting of cross-sectional studies.

### Outcomes

The primary outcomes were hypertension self-care behavior and self-efficacy, which are the 2 central components of hypertension self-management.^2^ Both outcomes were assessed at baseline using the Hypertension Self-Care Profile scale, which is a validated tool for measuring hypertension self-care.^3,16^ Participants self-reported hypertension self-care on a 4-point Likert scale in response to questions about 20 behaviors that are critical for BP control. The Hypertension Self-Care score was derived as the sum of the 20 items for each of the behavior (eTable 1 in Supplement 1) and efficacy (eTable 2 in Supplement 1) outcomes (range for scores, 20-80 [higher scores signify better hypertension self-care]).

### Exposures

Our primary exposure variable was participant’s self-reported neighborhood health score (hereafter referred to as perceived neighborhood health). This was measured at baseline using a questionnaire adapted from the neighborhood environment scale that has been validated for measuring constructs relevant to hypertension control, such as access to physical activity resources.^17^ This adapted questionnaire assessed 4 domains of the neighborhood environment scale: aesthetic quality (6 questions), walkability (10 questions), safety (3 questions), and violence (4 questions) (eTable 3 in Supplement 1). The means of the items were determined within each domain to obtain domain-specific scores for each participant, in which a higher score on each domain signified better perceived neighborhood health. We computed an overall neighborhood health score by converting the raw scores to domain-specific *z* scores, then averaging across the 4 *z* scores.

### Potential Modifiers

We evaluated ADI and healthy food availability at home as potential modifiers of the association of perceived neighborhood health with the Hypertension Self-Care Profile. The ADI level was ascertained through linkage of participants’ geocoded home addresses to the publicly available ADI data set for Maryland.^18,19^ The 2015 ADI data were used in this study as data from earlier years were no longer available at the time of data acquisition. We used national ranking to assign ADI percentiles to participants based on the US Census Block Group affiliated with the participant’s geocoded residential address. ADI is composed of 17 measures of the neighborhood socioeconomic environment across the subdomains of education, income/employment, housing quality, and household characteristics (eTable 4 in Supplement 1).^19^ Higher percentiles corresponded to more deprivation. Healthy food availability at home was assessed at baseline based on direct inspection by the study team of available food items in participants’ homes. In affirmative responses to the question, “Do you have [food item] in your home?,” participants were asked to show a trained research staff member the food item for confirmation. The assessment focused on food groups that are recommended for BP control and included fresh or frozen vegetables; fresh or frozen fruits; low- or no-fat dairy products; whole grains; nuts, seeds, and beans; and cooking oil. Responses were scored 1 if the trained research staff member confirmed that the food item was in the home and scored 0 if the food item was not confirmed to be in the home. The scores were summed within these food groups and converted to *z* scores. The healthy food availability at home index was then computed by averaging the food group–specific *z* scores.

In a post hoc analysis, we evaluated in-home availability of kitchen appliances as an additional potential effect modifier. This was assessed at baseline based on direct inspection in participants’ homes by the study team member. The assessment focused on 5 kitchen appliances that are essential for preparation and storage of foods that are recommended for BP control: refrigerator, oven, microwave oven, stovetop, and freezer. Responses to each kitchen appliance availability were scored 1 if the trained research staff member confirmed that the kitchen appliance was in the home or 0 if absent. We summed the raw scores for the 5 appliances (score range, 0-5) and converted the raw scores to *z* scores.

### Covariates

Additional variables of interest included demographic characteristics: age; sex; poverty, defined using participant-reported household income and number of individuals supported by the income and classified based on the 2015 Federal Poverty Guidelines^20^; educational level; and employment. Lifestyle and comorbid factors included tobacco use; estimated glomerular filtration rate; body mass index (calculated as weight in kilograms divided by height in meters squared); diabetes; physical activity score, defined as minutes of physical activity per week by summing participant-reported minutes spent walking and engaged in moderate- or vigorous-intensity activity^21^; depression, assessed using the Personal Health Questionnaire (PHQ) Depression Scale (score range, 0-24)^22^; perceived stress, measured using the Perceived Stress Scale^23^; resilience, assessed using the Resilience Brief Scale^24^; and BP control, defined as the mean of 3 measured BP levels greater than or equal to 140/90 mm Hg, which was the standard of care at the time of the ACT trial.

### Statistical Analysis

We report participants’ characteristics using counts and proportions for categorical variables and means (SDs) or medians (IQRs) for continuous variables. We compared participant characteristics across perceived neighborhood health subgroups using Mann-Whitney tests and Fisher exact tests as appropriate. No adjustment for multiple testing was made, as these comparisons were intended to be descriptive.

We fit generalized linear models regressing each outcome (ie, self-care behavior and self-efficacy) on perceived neighborhood health, adjusted for age, sex, education, and poverty. Psychosocial factors, eg, depression and perceived stress, were not included as adjustment covariates because these factors were mediators in our conceptual model for the association between neighborhood health and hypertension self-care.^25^ In separate models, we included an interaction term between perceived neighborhood health and ADI or healthy food availability at home to determine whether these were significant modifiers of the association between perceived neighborhood health and the outcomes. We used a more liberal significance level of 10%; if the interaction was not statistically significant at this level, we refit the model without the interaction term to obtain point estimates for the main association between perceived neighborhood health and hypertension self-care, additionally adjusted for ADI or healthy food availability. If the interaction effect was statistically significant, we obtained point estimates and the corresponding 95% CIs for the association of perceived neighborhood health with hypertension self-care outcomes at 1-SD increments of the modifier for illustrative purposes.

In secondary analyses, we used similar models as described above to examine the associations between each perceived neighborhood health subdomain and self-care behavior or self-efficacy and to assess for modification of the association by ADI or healthy food availability at home. In our post hoc analysis, we included an interaction term between perceived neighborhood health and kitchen appliances score to the above hypertension self-care models to evaluate additional potential modification of this association.

We performed analyses using SAS software, version 9.4 (SAS Institute Inc) and R, version 4.0.2 (R Foundation for Statistical Computing), and all hypothesis tests were 2-sided at the 5% significance level except for the above-named tests used for interactions. The analyses were conducted from August 5, 2021, to January 28, 2022.

## Results

### Participant Characteristics

All 159 participants in the parent ACT trial had complete data available for this analysis. Among these participants, median age was 57 (IQR, 49-64) years, mean (SD) age was 57 (11) years, 117 were women (74%) were women, 42 were men (26%), 11 (7%) had some college or higher educational level, 51 (32%) were working full time or part time, and 70 (44%) were living near or in poverty. Median body mass index was 33 (IQR, 29-39), median estimated glomerular filtration rate was 85 (IQR, 66-104) mL/min per 1.73 m^2^, and 76 individuals (48%) had diabetes (Table 1).

**Table 1.** Characteristics of Participants in the ACT Study

Variable	Overall	Median Neighborhood Health Score		*P* value
		Below	Above	
No. of participants	159	80 (50)	79 (50)	
Demographic characteristics				
Age, median (IQR), y	57 (49-64)	56 (48-61)	58 (52-66)	.14
Sex				
Men	42 (26)	22 (27)	20 (25)	.86
Women	117 (74)	58 (73)	59 (75)	
Poverty				
None	75 (47)	36 (45)	39 (49)	.13
Near or in poverty	70 (44)	40 (50)	30 (38)	
Not reported	14 (9)	4 (5)	10 (13)	
Educational level				
Less than high school diploma	62 (39)	37 (46)	25 (32)	.16
High school diploma	86 (54)	38 (48)	48 (61)	
Some college or higher	11 (7)	5 (6)	6 (7)	
Employment				
Working full or part time	51 (32)	21 (26)	30 (38)	.52
Unemployed	18 (11)	10 (13)	8 (10)	
Student or homemaker	11 (7)	6 (8)	5 (6)	
Retired or health issues	78 (49)	42 (53)	36 (46)	
Unknown	1 (1)	1 (1)	0	
Lifestyle and comorbidities				
Tobacco use				
Current	60 (38)	39 (49)	21 (27)	.02
Former	38 (24)	16 (20)	22 (28)	
Never	61 (38)	25 (31)	36 (45)	
eGFR, median (IQR), mL/min/1.73 m^2^	85 (66-104)	85 (66-104)	83 (66-104)	.77
BMI, median (IQR)	33 (29-39)	32 (28- 38)	34 (30-40)	.13
Diabetes	76 (48)	41 (51)	35 (44)	.43
Physical activity score, median (IQR)	438 (0-1272)	360 (0-964)	480 (0-1469)	.44
Depression, score, median (IQR)	5 (1-10)	6 (3-11)	4 (1-7)	<.001
Range	0-24			
Perceived stress score, median (IQR)	18 (13-21)	20 (15-22)	16 (10-21)	<.001
Range	14-56			
Resilience score (range, 10-70), median (IQR)	66 (60-70)	64 (60-69)	67 (61-70)	.03
Neighborhood environment				
Neighborhood health score, standardized^a^	0 (0.8)	−0.6 (0.5)	0.6 (0.4)	NA
Walkability	3.3 (0.6)	2.9 (0.4)	3.8 (0.4)	NA
Aesthetic quality	3.3 (0.8	2.7 (0.6)	3.9 (0.5)	NA
Safety	2.8 (0.8)	2.3 (0.7)	3.3 (0.6)	
Violence	3.2 (0.8)	2.8 (0.9)	3.6 (0.5)	NA
ADI, median (IQR)	81 (64-91)	86 (73-93)	73 (63-88)	<.001
In-home environment^b^				
Healthy food availability	0 (0.64)	−0.06 (0.66)	0.06 (0.63)	.18
Kitchen appliances availability				
*z* Score	0 (1)	−0.16 (1.14)	0.17 (0.81)	.04
No. of items, median (IQR)	5 (5-5)	5 (4-5)	5 (5-5)	.04
No. of items per household				
5	127 (80)	59 (74)	68 (86)	.12
4	9 (6)	4 (5)	5 (6)	
3	3 (2)	3 (4)	0	
2	1 (1)	1 (1)	0	
1	2 (1)	1 (1)	1 (1)	
0	17 (11)	12 (15)	5 (6)	
Proportion with each item				
Refrigerator	141 (89)	67 (84)	74 (94)	.08
Oven	134 (84)	63 (79)	71 (90)	.08
Microwave oven	135 (85)	64 (80)	71 (90)	.12
Stovetop	135 (85)	63 (79)	72 (91)	.04
Freezer	139 (87)	66 (83)	73 (92)	.09
Hypertension self-care^c^				
Self-care behavior				
Range	20-80			
Score, median (IQR)	50 (45-56)	49 (43-55)	51 (46-58)	.06
Score >60	20 (13)	5 (6)	15 (19)	.02
Self-efficacy				
Range	20-80			
Score, median (IQR)	64 (57-72)	61 (54-67)	68 (59-73)	.02
Score >60	93 (58)	42 (53)	51 (65)	.15
BP, median (IQR), mm Hg				
Systolic	137 (125-152)	135 (125-153)	138 (126-151)	.81
Diastolic	80 (72-89)	81 (75-89)	80 (72-87)	.22
BP control				
JNC 8: BP<130/80 mm Hg	80 (50)	42 (53)	38 (48)	.64

Abbreviations: ACT, Achieving Blood Pressure Control Together; ADI, area deprivation index; BMI, body mass index (calculated as weight in kilograms divided by height in meters squared); BP, blood pressure; eGFR, estimated glomerular filtration rate; JNC 8, Eighth Joint National Committee; NA, not applicable.

^a^ Neighborhood health was assessed across the 4 domains of aesthetic quality (6 questions), walkability (10 questions), safety (3 questions), and violence (4 questions). Raw scores were standardized within each domain using *z* scores and then averaged to derive the overall score.

^b^ Availability of healthy food and kitchen appliances at home was assessed based on direct inspection by the study team of available items in participants’ homes. Raw scores from responses were standardized for each variable using *z* scores.

^c^ Hypertension self-care was assessed on a 4-point Likert scale (1-4) in response to questions about 20 behaviors that are critical for BP control.

### Residential Characteristics

#### Perceived Neighborhood Health

The calculated mean (SD) perceived neighborhood health score reported by participants was 0 (0.8). Participants reporting high and low perceived neighborhood health had similar demographic characteristics. However, participants reporting higher vs lower perceived neighborhood health were less often current smokers (27% vs 49%); tended to have a more favorable psychosocial profile, with lower median depression (PHQ score, 4 vs 6) and perceived stress (16 vs 20) and higher resilience (67 vs 64); had a lower median ADI level (73 vs 86); and had a higher kitchen appliance score (0.17 vs −0.16) (Table 1).

#### Potential Modifiers

Median (IQR) ADI level was at the 81st percentile (IQR, 64-91 percentile). The calculated mean (SD) healthy food availability at home score was 0 (0.64), and the mean (SD) kitchen appliances score was 0 (1). Most participants had all 5 kitchen appliances available at home (127 [80%]); however, 17 participants (11%) did not have at least 1 kitchen appliance available. Participants reporting higher vs lower perceived neighborhood health were generally more likely to have kitchen appliances available at home: stovetop (72 [91%] vs 63 [79%]), oven (71 [90%] vs 63 [79%]), refrigerator (74 [94%] vs 67 [84%]), and freezer (73 [92%] vs 66 [83%]) (Table 1).

### BP and Hypertension Self-care Factors

Participants’ median hypertension self-care behavior score was 50 (IQR, 45-56), and only 20 participants (13%) had a score above 60 (range, 20-80). Median [IQR] hypertension self-care self-efficacy was 64 (IQR, 57-72) and 93 participants (58%) had a score greater than 60 (range, 20-80). Overall, only 80 participants (50%) had their BP controlled. Participants with higher vs lower neighborhood health scores had similar median systolic BP (138 vs 135 mm Hg), diastolic BP (80 vs 81 mm Hg), or BP control (48% vs 53%). Compared with participants reporting lower neighborhood health, those reporting better neighborhood health had a slightly higher median self-care behavior score (51 vs 49), were more likely to have a score greater than 60 (19% vs 6%), had a higher median self-efficacy score (68 vs 61), and were more likely to have a score greater than 60 (65% vs 53%) (Table 1).

### Associations of Perceived Neighborhood Health With Hypertension Self-care

Better perceived neighborhood health was associated with greater hypertension self-care behavior and self-efficacy. These associations were not significantly modified by ADI, but there was evidence that healthy food availability at home modified some of the associations, as described below.

#### ADI Levels

There were no significant interactions between perceived neighborhood health and ADI on hypertension self-care behavior (*P* = .74 for interaction) or self-efficacy (*P* = .85 for interaction). In multivariable main effects models additionally adjusted for ADI, higher perceived neighborhood health was associated with greater hypertension self-care behavior (β, 2.48; 95% CI, 0.63-4.33) and self-efficacy (β, 4.42; 95% CI, 2.25-6.59). These associations persisted for all neighborhood health subdomains except aesthetic quality (Table 2).

**Table 2.** ADI Level and Perceived Neighborhood Health Association With Hypertension Self-care Behavior and Self-efficacy

Variable	Hypertension			
	Self-care behavior (n = 157)^a^		Self-efficacy (n = 157)	
	β (95% CI)	*P* value for interaction	β (95% CI)	*P* value for interaction
Neighborhood health score	2.48 (0.63 to 4.33)	.74	4.42 (2.25 to 6.59)	.85
Neighborhood health subdomains^b^				
Aesthetic quality	1.42 (−0.37 to 3.21)	.49	2.34 (0.19 to 4.50)	.83
Walkability	2.68 (0.33 to 5.03)	.60	5.16 (2.40 to 7.93)	.31
Safety	1.91 (0.17 to 3.65)	.63	3.78 (1.73 to 5.83)	.65
Violence (n = 155)	1.85 (0.04 to 3.67)	.69	2.91 (0.73 to 5.09)	.45
Area deprivation index	0.03 (−0.06 to 0.12)	NA	0.03 (−0.07 to 0.13)	NA

Abbreviations: ADI, area deprivation index; NA, not applicable.

^a^ Hypertension self-care was assessed on a 4-point Likert scale (1-4) in response to questions about 20 behaviors that are critical for blood pressure control.

^b^ Neighborhood health was assessed across the 4 domains of aesthetic quality (6 questions), walkability (10 questions), safety (3 questions), and violence (4 questions). Raw scores were standardized within each domain using z scores and then averaged to derive the overall score.

#### Healthy Food Availability at Home

There were no significant interactions between perceived neighborhood health and healthy food availability at home on hypertension self-care behavior (*P* = .93 for interaction) or self-efficacy (*P* = .27 for interaction). In multivariable main effects models additionally adjusted for healthy food availability at home, better perceived neighborhood health was associated with greater hypertension self-care behavior (β, 2.46; 95% CI, 0.68-4.23) and self-efficacy (β, 4.08; 95% CI, 1.96-6.20) (Table 3). However, significant interactions were observed for the neighborhood health subdomains of aesthetic quality and violence between healthy food availability at home and hypertension self-care behavior. Specifically, the magnitude for the association of better perceived neighborhood aesthetic quality with greater hypertension self-care behavior was higher among participants with greater healthy food availability at home: β for −1 SD, −0.29 (95% CI, −2.89 to 2.30); β for 0 SD, 1.34 (95% CI, −0.39 to 3.06); β for 1 SD, 2.97 (95% CI, 0.46-5.47); and β for 2 SDs, 4.60 (0.53-8.67) (*P* = .09 for interaction) (Table 4).

**Table 3.** Healthy Food Availability at Home and Association of Perceived Neighborhood Health With Hypertension Self-care Behavior

Variable	Hypertension			
	Self-care behavior (n = 159)^a^		Self-efficacy (n = 159)	
	β (95% CI)	*P* value for interaction	β (95% CI)	*P* value for interaction
Neighborhood health score	2.46 (0.68 to 4.23)	.93	4.08 (1.96 to 6.20)	.27
Neighborhood health subdomains^b^				
Aesthetic quality	NA	.09	2.36 (0.23 to 4.49)	.67
Walkability	2.88 (0.61 to 5.15)	.82	4.94 (2.22 to 7.66)	.35
Safety	1.76 (0.08 to 3.44)	.51	3.35 (1.34 to 5.36)	>.99
Violence	NA	.04	2.71 (0.56 to 4.85)	.34
Healthy food availability at home^c^	1.81 (−0.33 to 3.95)	NA	0.98 (−1.58 to 3.55)	NA

Abbreviation: NA, not applicable.

^a^ Hypertension self-care was assessed on a 4-point Likert scale (1-4) in response to questions about 20 behaviors that are critical for blood pressure control.

^b^ Neighborhood health was assessed across the 4 domains of aesthetic quality (6 questions), walkability (10 questions), safety (3 questions), and violence (4 questions). Raw scores were standardized within each domain using z scores and then averaged to derive the overall score.

^c^ Availability of healthy food and kitchen appliances at home was assessed based on direct inspection by the study team of available items in participants’ homes. Raw scores from responses were standardized for each variable using *z* scores.

**Table 4.** HFAH and Association of Perceived Neighborhood Aesthetic Quality and Violence With Hypertension Self-care Behavior

Variable	Hypertension self-care behavior (n = 159)	
	β (95% CI)	*P* value for interaction
**Aesthetic quality**		
HFAH score at −1 SD	−0.29 (−2.89 to 2.30)	.09
HFAH score at 0 SD, mean	1.34 (−0.39 to 3.06)	
HFAH score at 1 SD	2.97 (0.46 to 5.47)	
HFAH score at 2 SDs	4.60 (0.53 to 8.67)	
**Violence**		
HFAH score at −1 SD	3.69 (1.31 to 6.08)	.04
HFAH score at 0 SD, mean	1.85 (0.13 to 3.57)	
HFAH score at 1 SD	0.01 (−2.53 to 2.54)	
HFAH score at 2 SDs	−1.83 (−5.84 to 2.18)	

Abbreviation: HFAH, healthy food availability at home.

Conversely, the association of worse perceived neighborhood violence with lower hypertension self-care behavior was increasingly attenuated at higher healthy food availability at home scores, suggesting that healthy food availability at home had a graded protective effect on hypertension self-care behavior for participants reporting worse neighborhood violence scores: β for −1 SD, 3.69 (95% CI, 1.31-6.08); β for 0 SD, 1.85 (95% CI, 0.13-3.57); β for 1 SD, 0.01 (95% CI, −2.53 to 2.54); and β for 2 SDs, −1.83 (−5.84 to 2.18) (*P* = .04 for interaction) (Table 4). In post hoc analyses, there was no statistically significant interaction between perceived neighborhood health and the presence of kitchen appliances with hypertension self-care behavior (*P* = .84 for interaction) or with self-efficacy (*P* = .99 for interaction) (eTable 5 in Supplement 1).

## Discussion

Among Black individuals with social disadvantages who had hypertension, perceptions of better neighborhood health, including walkability, aesthetic quality, safety, and violence, were associated with greater hypertension self-care behavior and self-efficacy, even after adjustment for age, sex, educational level, and poverty. This association was not significantly modified by ADI—a census-based socioeconomic assessment of the neighborhood (ie, US Census block group). However, the magnitude of associations of better perceived neighborhood health, in particular, the subdomains of better aesthetic quality and lower violence, with greater hypertension self-care behavior were higher among participants with greater healthy food availability at home. These findings suggest that contextual neighborhood features and in-home resources might play a role in hypertension self-care.

Few published studies have investigated the association between patient-described neighborhood characteristics and hypertension self-management. Prior studies have predominantly examined and reported associations of census-based neighborhood assessments, such as ADI, with hypertension risk^8,9^ and suboptimal BP control.^10^ ADI and other census-based neighborhood measures assess structural socioeconomic constructs, eg, neighborhood median household income, educational attainment, housing quality, and poverty.^16^ However, it has been suggested that the observed association of these neighborhood socioeconomic factors with health outcomes, including BP control, is mediated by additional, less recognized constructs, such as neighborhood walkability and safety.^12^ We found evidence linking these additional neighborhood constructs, including walkability, aesthetic quality, and safety, with hypertension self-care behavior and self-efficacy. There was no interaction between these patient self-described neighborhood characteristics and ADI and hypertension self-care, which suggests that hypertension self-management might be independent of existing census-based neighborhood socioeconomic features. Thus, future person-centered interventions might need to engage community partners who can self-identify neighborhood factors for intervention targets to optimize mitigating contextual barriers to hypertension self-management.

In our study, the magnitude of association of better perceived neighborhood health with greater hypertension self-care behavior was significantly higher among individuals with greater availability of food items at home for BP control. This synergism underscores the importance of simultaneously addressing contextual neighborhood and in-home resources to optimize hypertension self-management. For instance, individuals residing in neighborhoods with high levels of violence often have fewer healthy food outlets available in the neighborhood due to structural barriers, including disinvestment,^26^ and consequently might attempt trips to grocery stores only during perceived safe hours^27,28^; either instance would limit access to food resources at home for hypertension self-care.^29,30^ Likewise, individuals with limited availability of food resources at home might be less motivated to use neighborhood resources, such as parks, for physical activity. We found no evidence suggesting that the availability of kitchen appliances in the home might be associated with perceived neighborhood health and hypertension self-care. However, this lack of a synergistic interaction could be because most patients in this cohort had kitchen appliances available at home. Furthermore, the few participants who did not have kitchen appliances were also more likely to report poor neighborhood health. Future studies are needed to better understand the complex interplay of in-home vs external resources to support hypertension self-management.

### Strengths and Limitations

This study has strengths. The data on self-reported measures, such as hypertension self-care and neighborhood health, were obtained using validated instruments. Furthermore, the study investigators conducted home visits to confirm the availability of healthy food resources in participants’ homes. To our knowledge, this study is one of the first to assess the availability of healthy food and kitchen appliances in participants’ homes via direct, standardized inspections by trained study team members, which minimizes biases inherent in self-reports.

This study has limitations. First, the ACT study was conducted among a select population of Black individuals with social disadvantages and hypertension and therefore may lack generalizability. The small sample size of the present study (n = 159 participants) also limits generalizability of the findings. Nonetheless, few studies have explored potential associations between perceived neighborhood health and BP self-management specifically among Black individuals.

## Conclusions

In this cross-sectional study, better perceived neighborhood health was associated with greater hypertension self-care behavior and self-efficacy among Black individuals with hypertension in socially disadvantaged settings. In-home food availability had an association with hypertension self-care behavior among individuals reporting worse neighborhood health. Interventions to improve hypertension self-management, particularly among individuals with social disadvantages, might require multifaceted interventions targeting both patients’ self-described contextual barriers to self-care and the availability of healthy food resources in the home.

## Notes

Supplement 1. **eTable 1.** Questionnaire Items in the Hypertension Self-Care Behavior Instrument **eTable 2.** Questionnaire Items in the Hypertension Self-Efficacy Instrument **eTable 3.** Questionnaire Items in the Neighborhood Health Score Behavior Instrument **eTable 4.** Neighborhood Socioeconomic Measures Included in the Area Deprivation Index (ADI) **eTable 5.** Availability of Kitchen Appliances Did Not Modify the Association of Patient-Described Neighborhood Health With Hypertension Self-Care Behavior

Supplement 2.
Data Sharing Statement

## References

[1] Whelton PK, Carey RM, Aronow WS, . 2017 ACC/AHA/AAPA/ABC/ACPM/AGS/APhA/ASH/ASPC/NMA/PCNA guideline for the prevention, detection, evaluation, and management of high blood pressure in adults: a report of the American College of Cardiology/American Heart Association Task Force on Clinical Practice Guidelines. J Am Coll Cardiol. 2018;71(19):e127-e248. doi:10.1016/j.jacc.2017.11.006 29146535

[2] Bodenheimer T, Lorig K, Holman H, Grumbach K. Patient self-management of chronic disease in primary care. JAMA. 2002;288(19):2469-2475. doi:10.1001/jama.288.19.2469 12435261

[3] Han HR, Lee H, Commodore-Mensah Y, Kim M. Development and validation of the Hypertension Self-Care Profile: a practical tool to measure hypertension self-care. J Cardiovasc Nurs. 2014;29(3):E11-E20. doi:10.1097/JCN.0b013e3182a3fd46 24088621 PMC3972381

[4] Boulware LE, Ephraim PL, Hill-Briggs F, . Hypertension self-management in socially disadvantaged African Americans: the Achieving Blood Pressure Control Together (ACT) randomized comparative effectiveness trial. J Gen Intern Med. 2020;35(1):142-152. doi:10.1007/s11606-019-05396-7 31705466 PMC6957583

[5] Bosworth HB, Powers BJ, Olsen MK, . Home blood pressure management and improved blood pressure control: results from a randomized controlled trial. Arch Intern Med. 2011;171(13):1173-1180. doi:10.1001/archinternmed.2011.276 21747013

[6] Maciejewski ML, Bosworth HB, Olsen MK, . Do the benefits of participation in a hypertension self-management trial persist after patients resume usual care? Circ Cardiovasc Qual Outcomes. 2014;7(2):269-275. doi:10.1161/CIRCOUTCOMES.113.000309 24619321

[7] Li R, Liang N, Bu F, Hesketh T. The effectiveness of self-management of hypertension in adults using mobile health: systematic review and meta-analysis. JMIR Mhealth Uhealth. 2020;8(3):e17776. doi:10.2196/17776 32217503 PMC7148553

[8] Claudel SE, Adu-Brimpong J, Banks A, . Association between neighborhood-level socioeconomic deprivation and incident hypertension: a longitudinal analysis of data from the Dallas Heart Study. Am Heart J. 2018;204:109-118. doi:10.1016/j.ahj.2018.07.005 30092412 PMC6217793

[9] Xu J, Lawrence KG, O’Brien KM, Jackson CL, Sandler DP. Association between neighbourhood deprivation and hypertension in a US-wide cohort. J Epidemiol Community Health. 2022;76(3):268-273. doi:10.1136/jech-2021-216445 34789553 PMC8837699

[10] Durfey SNM, Kind AJH, Buckingham WR, DuGoff EH, Trivedi AN. Neighborhood disadvantage and chronic disease management. Health Serv Res. 2019;54(suppl 1):206-216. doi:10.1111/1475-6773.13092 30468015 PMC6341202

[11] McDoom MM, Cooper LA, Hsu YJ, Singh A, Perin J, Thornton RLJ. Neighborhood environment characteristics and control of hypertension and diabetes in a primary care patient sample. J Gen Intern Med. 2020;35(4):1189-1198. doi:10.1007/s11606-020-05671-y 32043258 PMC7174485

[12] Mujahid MS, Diez Roux AV, Morenoff JD, . Neighborhood characteristics and hypertension. Epidemiology. 2008;19(4):590-598. doi:10.1097/EDE.0b013e3181772cb2 18480733

[13] Diez Roux AV. Neighborhoods and health: what do we know? what should we do? Am J Public Health. 2016;106(3):430-431. doi:10.2105/AJPH.2016.303064 26885960 PMC4815954

[14] Christie CD, Friedenreich CM, Vena JE, Turley L, McCormack GR. Cross-sectional and longitudinal associations between the built environment and walking: effect modification by socioeconomic status. BMC Public Health. 2022;22(1):1233. doi:10.1186/s12889-022-13611-0 35729509 PMC9210749

[15] Ephraim PL, Hill-Briggs F, Roter DL, . Improving urban African Americans’ blood pressure control through multi-level interventions in the Achieving Blood Pressure Control Together (ACT) study: a randomized clinical trial. Contemp Clin Trials. 2014;38(2):370-382. doi:10.1016/j.cct.2014.06.009 24956323 PMC4169070

[16] Ma Y, Cheng HY, Sit JWH, Chien WT. Psychometric evaluation of the Chinese version of hypertension self-care profile. J Cardiovasc Nurs. 2021;36(5):420-429. doi:10.1097/JCN.0000000000000708 32590612

[17] Echeverria SE, Diez-Roux AV, Link BG. Reliability of self-reported neighborhood characteristics. J Urban Health. 2004;81(4):682-701. doi:10.1093/jurban/jth151 15466849 PMC3455923

[18] Kind AJH, Buckingham WR. Making neighborhood-disadvantage metrics accessible—the neighborhood atlas. N Engl J Med. 2018;378(26):2456-2458. doi:10.1056/NEJMp1802313 29949490 PMC6051533

[19] Maroko AR, Doan TM, Arno PS, Hubel M, Yi S, Viola D. Integrating social determinants of health with treatment and prevention: a new tool to assess local area deprivation. Prev Chronic Dis. 2016;13:E128. doi:10.5888/pcd13.160221 27634778 PMC5027849

[20] Office of the Assistant Secretary for Planning and Evaluation, US Department of Health and Human Services. 2015 Poverty guidelines. Accessed February 12, 2022. https://aspe.hhs.gov/2015-poverty-guidelines

[21] Porch TC, Bell CN, Bowie JV, . The role of marital status in physical activity among African American and White men. Am J Mens Health. 2016;10(6):526-532. doi:10.1177/1557988315576936 25804218 PMC5809331

[22] Kroenke K, Spitzer RL, Williams JB. The PHQ-9: validity of a brief depression severity measure. J Gen Intern Med. 2001;16(9):606-613. doi:10.1046/j.1525-1497.2001.016009606.x 11556941 PMC1495268

[23] Cohen S, Kamarck T, Mermelstein R. A global measure of perceived stress. J Health Soc Behav. 1983;24(4):385-396. doi:10.2307/2136404 6668417

[24] Smith BW, Dalen J, Wiggins K, Tooley E, Christopher P, Bernard J. The Brief Resilience Scale: assessing the ability to bounce back. Int J Behav Med. 2008;15(3):194-200. doi:10.1080/10705500802222972 18696313

[25] Anderson CE, Broyles ST, Wallace ME, Bazzano LA, Gustat J. Association of the neighborhood built environment with incident and prevalent depression in the rural South. Prev Chronic Dis. Published online July 8, 2021. doi:10.5888/pcd18.200605 PMC8269752 34237245

[26] Tung EL, Boyd K, Lindau ST, Peek ME. Neighborhood crime and access to health-enabling resources in Chicago. Prev Med Rep. 2018;9:153-156. doi:10.1016/j.pmedr.2018.01.017 29527469 PMC5840856

[27] Hilmers A, Hilmers DC, Dave J. Neighborhood disparities in access to healthy foods and their effects on environmental justice. Am J Public Health. 2012;102(9):1644-1654. doi:10.2105/AJPH.2012.300865 22813465 PMC3482049

[28] Rummo PE, Guilkey DK, Ng SW, Popkin BM, Evenson KR, Gordon-Larsen P. Beyond supermarkets: food outlet location selection in four US cities over time. Am J Prev Med. 2017;52(3):300-310. doi:10.1016/j.amepre.2016.08.042 27865651 PMC5448705

[29] Cohen L, Davis R, Lee V, Valdovinos E. Addressing the intersection: preventing violence and promoting healthy eating and active living. May 2010. Accessed February 12, 2022. https://www.preventioninstitute.org/sites/default/files/publications/VNPA_Addressing%20the%20Intersection_051810.pdf

[30] Madrigal JM, Cedillo-Couvert E, Ricardo AC, ; CRIC Study Investigators. Neighborhood food outlet access and dietary intake among adults with chronic kidney disease: results from the Chronic Renal Insufficiency Cohort Study. J Acad Nutr Diet. 2020;120(7):1151-1162.e3. doi:10.1016/j.jand.2019.12.013 32146126 PMC7321867

