# Supplementary material for: Association of Perceived Neighborhood Health With Hypertension Self-care
Source: JAMA Netw Open. 2023 Feb 10;6(2):e2255626. doi: 10.1001/jamanetworkopen.2022.55626 (PMC9918870; doi:10.1001/jamanetworkopen.2022.55626)
Supplement: Supplement 1. — eTable 1. Questionnaire Items in the Hypertension Self-Care Behavior Instrument eTable 2. Questionnaire Items in the Hypertension Self-Efficacy Instrument eTable 3. Questionnaire Items in the Neighborhood Health Score Behavior Instrument eTable 4. Neighborhood Socioeconomic Measures Included in the Area Deprivation Index (ADI) eTable 5. Availability of Kitchen Appliances Did Not Modify the Association of Patient-Described Neighborhood Health With Hypertension Self-Care Behavior [file jamanetwopen-e2255626-s001.pdf]

Supplementary Online Content

Lunyera J, Davenport CA, Ephraim P, et al. Association of perceived neighborhood health with hypertension self-care. *JAMA Netw Open*. 2023;6(2):e2255626. doi:10.1001/jamanetworkopen.2022.55626

eTable 1. Questionnaire Items in the Hypertension Self-Care Behavior Instrument

eTable 2. Questionnaire Items in the Hypertension Self-Efficacy Instrument

eTable 3. Questionnaire Items in the Neighborhood Health Score Behavior Instrument

eTable 4. Neighborhood Socioeconomic Measures Included in the Area Deprivation Index (ADI)

eTable 5. Availability of Kitchen Appliances Did Not Modify the Association of Patient-Described Neighborhood Health With Hypertension Self-Care Behavior

This supplementary material has been provided by the authors to give readers additional information about their work.

© 2023 Lunyera J et al. *JAMA Network Open.*

eTable 1: Questionnaire items in the hypertension self-care behavior instrument

Items	Questionnaire prompts*
1	How often do you take part in regular physical activity, for example, 30 minutes of brisk walking 4-5 times a week?
2	How often do you read nutrition facts labels to check information on sodium content?
3	How often do you replace traditional high-salt foods, such as canned soups or Oodles of Noodles, with low-salt products, such as homemade soups or fresh vegetables?
4	How often do you limit use of high-salt condiments, for example ketchup?
5	How often do you eat less than 1 teaspoon or 6 grams of salt per day?
6	How often do you eat less foods that are high in saturated and trans-fat, for example red meat, butter, shortening, or lard?
7	How often do you use broil, bake or steam instead of frying when cooking?
8	How often do you read nutrition facts labels to check information on saturated and trans-fat for example in butter, red meat, lard, or shortening?
9	How often do you replace traditional high-fat foods such as deep-fried chicken with low-fat products such as baked chicken?
10	How often do you limit total calorie intake from fat to less than 65 grams daily?
11	How often do you eat 5 or more servings of fruits and vegetables daily?
12	How often do you practice moderation in drinking alcohol daily - 2 glasses or less for men; 1 glass or less for women?
13	How often do you practice non-smoking?
14	How often do you check your blood pressure at home?
15	How often do you take your high blood pressure medicine regularly?
16	How often do you get your prescriptions filled?
17	How often do you keep your weight down?
18	How often do you monitor situations that cause a high level of stress resulting in blood pressure elevation, for example arguments or a death in the family?
19	How often do you engage in activities that can lower stress such as deep breathing or meditation?
20	How often do you see a doctor regularly?
*Possible responses: “not confident” (score =1); “somewhat confident” (score =2); “confident” (score =3); and “very confident” (score =4). The hypertension self-care behavior score was derived as the sum of responses to the above 20 questionnaire items.	

© 2023 Lunyera J et al. *JAMA Network Open.*

eTable 2: Questionnaire items in the hypertension self-efficacy instrument

Items	Questionnaire prompts*
1	How confident are you that you could take part in regular physical activity, for example 30 minutes of brisk walking 4-5 times a week?
2	How confident are you that you could eat less processed foods such as lunch meats, canned or frozen foods?
3	How confident are you that you could read nutrition facts labels to check information on sodium content?
4	How confident are you that you could replace traditional high-salt foods such as canned soups or Oodles of Noodles with low-salt products such as homemade soups or fresh vegetables?
5	How confident are you that you could limit use of high-salt condiments (for example, ketchup)?
6	How confident are you that you could eat less than 1 teaspoon or 6 grams of table salt per day?
7	How confident are you that you could eat less foods that are high in saturated and trans-fat (for example, red meat, butter, lard, or shortening)?
8	How confident are you that you could broil, bake or steam instead of frying when cooking?
9	How confident are you that you could read nutrition facts labels to check information on saturated and trans-fat, for example in butter, red meats, lard, or shortening?
10	How confident are you that you could replace traditional high-fat foods such as deep-fried chicken with low-fat products such as baked chicken?
11	How confident are you that you could limit total calorie intake from fat to less than 65grams daily?
12	How confident are you that you could eat 5 or more servings of fruits and vegetables daily?
13	How confident are you that you could practice moderation in drinking alcohol daily - 2 glasses or less for men; 1 glass or less for women?
14	How confident are you that you could practice non-smoking?
15	How confident are you that you could check your blood pressure at home?
16	How confident are you that you could take your high blood pressure medication(s)?
17	How confident are you that you could get your prescriptions filled?
18	How confident are you that you could keep your weight down?
19	How confident are you that you could try to stay away from anything and anybody that causes any kind of stress?
20	How confident are you that you could see a doctor regularly?
*Possible responses: “not confident” (score =1); “somewhat confident” (score =2); “confident” (score =3); and “very confident” (score =4). The hypertension self-efficacy score was derived as the sum of responses to the above 20 questionnaire items.	

© 2023 Lunyera J et al. *JAMA Network Open.*

eTable 3: Questionnaire items in the neighborhood health score instrument

Questionnaire items and prompts by neighborhood health subdomains	
Aesthetic Quality*	
1	My neighborhood is attractive
2	There is a lot of trash and litter on the street in my neighborhood
3	There are interesting things to do in my neighborhood
4	There is a lot of noise in my neighborhood
5	In my neighborhood, the buildings and homes are well maintained
6	The buildings and houses in my neighborhood are interesting
Walkability*	
1	My neighborhood offers many opportunities to be physically active
2	Local sports clubs and other facilities in my neighborhood offer many opportunities to get exercise
3	It is pleasant to walk in my neighborhood
4	The trees in my neighborhood provide enough shade
5	My neighborhood has heavy traffic
6	There are busy roads to cross when out for walks in my neighborhood
7	In my neighborhood, it is easy to walk to places
8	There are stores within walking distance of my home
9	I often see other people walking in my neighborhood
10	I often see other people exercise in my neighborhood, for example jogging, bicycling, or playing sports
Safety*	
1	I feel safe walking in my neighborhood day or night
2	My neighborhood is safe from crime
3	Violence is a problem in my neighborhood
Violence in the past 6 months**	
1	During the past six months, how often was there a fight in your neighborhood in which a weapon was used? Weapons include anything used to attack a person, not just firearms
2	During the past six months, how often were there gang fights in your neighborhood?
3	During the past six months, how often was there a sexual assault or rape in your neighborhood?
4	During the past six months, how often was there a robbery or mugging in your neighborhood?
* Possible responses: “strongly disagree” (score =1); “disagree” (score =2); “neither agree nor disagree” (score =3); “agree” (score =4); and “strongly agree” (score =5)<br>** Possible responses: “never” (score =1); “rarely” (score =2); “sometimes” (score =3); and “often” (score =4)	

© 2023 Lunyera J et al. *JAMA Network Open.*

eTable 4: Neighborhood socioeconomic measures included in the area deprivation index (ADI)

ADI Domain	ADI Neighborhood Measure
Education	% Population aged 25 years or older with less than 9 years of education
	% Population aged 25 years or older with at least a high school diploma
	% Employed population aged 16 years or older in white-collar occupations
Income/employment	Median family income in US dollars
	Income disparity
	% Families below federal poverty level
	% Population below 150% of federal poverty level
	% Civilian labor force population aged 16 years and older who are unemployed
Housing quality	Median home value in US dollars
	Median gross rent in US dollars
	Median monthly mortgage in US dollars
	% Owner-occupied housing units
	% Occupied housing units without complete plumbing
Household characteristics	% Single-parent households with children younger than 18
	% Households without a motor vehicle
	% Households without a telephone
	% Households with more than 1 person per room

© 2023 Lunyera J et al. *JAMA Network Open.*

eTable 5: Availability of kitchen appliances did not modify the association of patient-described neighborhood health with hypertension self-care behavior

	Hypertension self-care behavior		Hypertension self-efficacy	
	P-value interaction	β (95% CI)	P-value interaction	β (95% CI)
No. of participants	159		159	
Neighborhood health score	0.84	2.50 (0.70 to 4.29)	0.99	4.07 (1.93 to 6.21)
Neighborhood health subdomains				
Aesthetic quality	0.38	1.47 (-0.29 to 3.24)	0.59	2.38 (0.25 to 4.51)
Walkability	0.85	2.77 (0.48 to 5.06)	0.39	4.84 (2.11 to 7.57)
Safety	0.47	1.87 (0.17 to 3.57)	0.76	3.37 (1.35 to 5.40)
Violence	0.85	1.89 (0.11 to 3.68)	0.16	2.60 (0.43 to 4.76)
Kitchen appliances availability	NA	0.0 (-0.87 to 0.87)	NA	0.12 (-0.91 to 1.15)

CI, confidence interval
Neighborhood health was assessed across the four domains of aesthetic quality (6 questions); walkability (10 questions); safety (3 questions); and violence (4 questions). Raw scores were standardized within each domain using z scores and then averaged to derive the overall score.
Availability of kitchen appliances at home was assessed based on direct inspection by study team of available items in participants' homes. Raw scores from responses were standardized using z scores.
Hypertension self-care was assessed on a 4-point Likert scale (1-4) in response to questions about 20 behaviors that are critical for blood pressure (BP) control.

© 2023 Lunyera J et al. *JAMA Network Open.*
