# Supplementary material for: Association of Perceived Neighborhood Health With Hypertension Self-care
Source: JAMA Netw Open. 2023 Feb 10;6(2):e2255626. doi: 10.1001/jamanetworkopen.2022.55626 (PMC9918870; doi:10.1001/jamanetworkopen.2022.55626)
Supplement: Supplement 2. — Data Sharing Statement [file jamanetwopen-e2255626-s002.pdf]

Data Sharing Statement

Lunyera. Association of Perceived Neighborhood Health With Hypertension Self-Care. *JAMA Netw Open*. Published February 10, 2023. doi:10.1001/jamanetworkopen.2022.55626

Data
Data available: No
